# Cerebrospinal fluid monoamine metabolite profiles in bipolar disorder, ADHD, and controls

**DOI:** 10.1007/s00702-017-1746-3

**Published:** 2017-06-27

**Authors:** Erik Pålsson, Carl Sellgren, Eleonore Rydén, Ruth Kizza, Aurimantas Pelanis, Henrik Zetterberg, Kaj Blennow, Mikael Landén

**Affiliations:** 1 Department of Psychiatry and Neurochemistry, Institute of Neuroscience and Physiology, Sahlgrenska Academy at University of Gothenburg, POB 431, 405 30 Göteburg, Sweden; 2 Section of Psychiatry, Department of Clinical Neuroscience, Karolinska Institutet, Stockholm, Sweden; 3 Sahlgrenska University Hospital, Göteburg, Sweden; 4 Clinical Neurochemistry Laboratory, Sahlgrenska University Hospital, Mölndal, Sweden; 5 Department of Molecular Neuroscience, UCL Institute of Neurology, Queen Square, London, UK; 6 Stanley Center for Psychiatric Research, Broad Institute of MIT and Harvard, Cambridge, MA USA; 7 Department of Psychiatry, Massachusetts General Hospital, Harvard Medical School, Boston, MA USA; 8 Department of Medical Epidemiology and Biostatistics, Karolinska Institutet, Stockholm, Sweden

**Keywords:** Bipolar disorder, ADHD, Cerebrospinal fluid, Dopamine, Serotonin, Norepinephrine, Monoamines

## Abstract

Alterations in monoaminergic signaling are suggested as key aspects of the pathophysiology in bipolar disorder and ADHD, but it is not known if the monoamine metabolic profile differs between these disorders. One method to study monoaminergic systems in humans is to measure monoamine end-point metabolite concentrations in cerebrospinal fluid (CSF). Here, we analyzed CSF monoamine metabolite concentrations in 103 adults with bipolar disorder, 72 adults with ADHD, and 113 controls. Individuals with bipolar disorder had significantly higher homovanillic acid (HVA, 264 ± 112 nmol/L, *p* < 0.001) and 5-hydroxyindoleacetic acid (5-HIAA, 116 ± 42 nmol/L, *p* = 0.001) concentration, but lower 3-methoxy-4-hydroxyphenylethyleneglycol (MHPG, 38 ± 8 nmol/L, *p* < 0.001) concentrations than controls (HVA, 206 ± 70 nmol/L; 5-HIAA, 98 ± 31 nmol/L; and MHPG, 42 ± 7 nmol/L). Higher HVA concentrations were associated with a history of psychosis in the bipolar disorder sample. Subjects with ADHD had higher HVA (240 ± 94 nmol/L, *p* < 0.001) concentrations compared with controls. In addition, SSRI treatment was associated with lower 5-HIAA concentrations in both patient groups. A power analysis indicated that for within-group comparisons, only large effects would be reliably detectable. Thus, there may be moderate-to-small effects caused by medication that were not detected due to the limited size of the sub-groups in these analyses. In conclusion, the present study suggests disorder-specific alterations of CSF monoamine metabolite concentrations in patients with bipolar disorder and ADHD compared with controls; these differences were independent of acute symptoms and medication effects.

## Introduction

According to the monoamine hypothesis of mood disorders, based on pharmacological studies from the end of the 1950s and onwards (Akiskal and McKinney 1973), alterations in monoaminergic signaling are key aspects of the pathophysiology in several psychiatric disorders. A central tenet is that depression is caused by reduced activity in the monoaminergic signaling systems and that symptoms are alleviated by increasing monoaminergic activity [although subsequent work paints a more complex picture (Owens 2004)]. Contrariwise, the hypothesis predicts that increased monoaminergic activity is the pathophysiological basis for mania (Tissot 1975). A monoaminergic hypothesis of attention-deficit/hyperactivity disorder (ADHD) was suggested in the early 1970s (Kornetsky 1970) and implicates a dysregulated noradrenergic, dopaminergic, and probably serotonergic, signaling (Biederman and Spencer 1999; Bonvicini et al. 2016). Despite tremendous efforts to probe pathophysiological aberrations in monoamine signaling in patients with mood disorders and ADHD, the nature of such changes remains unclear (Chaudhury et al. 2015). The strongest argument in support of this hypothesis is still that drugs targeting brain monoamine signaling are effective across several psychiatric disorders including schizophrenia, major depressive disorder, bipolar disorder, and ADHD.

One method to study the monoaminergic systems in humans is to measure the end-metabolites of dopamine (DA), serotonin (5-HT), and norepinephrine (NE) in cerebrospinal fluid (CSF). Sampling of CSF provides an indirect measure of brain monoamine concentrations as compared to the study of urine or blood that merely provides information on peripheral concentrations. Indeed, CSF concentrations of the DA metabolite homovanillinic acid (HVA) and the 5-HT metabolite 5-hydroxyindoleacetic acid (5-HIAA) correlate highly with the brain tissue concentrations in dogs (Moir et al. 1970). Moreover, experiments in rats have shown that CSF HVA and 5-HIAA concentrations are indicative of brain DA and 5-HT turnover (Nielsen and Moore 1982).

In support of the monoamine theory of mood disorders, low CSF concentrations of HVA and 5-HIAA have been associated with depressive symptoms in patients with mood disorders (Agren 1980; Kasa et al. 1982; Peabody et al. 1987; Asberg et al. 1984). Observations in patients with mania are inconsistent and report increased, decreased, or unchanged HVA and 5-HIAA concentrations (Goodwin and Ghaemi 1998; Shiah and Yatham 2000; Gerner et al. 1984; Swann et al. 1983). Furthermore, acute mania or atypical symptoms of depression have been associated with higher concentrations of the NE metabolite 3-methoxy-4-hydroxyphenylglycol (MHPG) in CSF from patients with a mood disorder (Swann et al. 1994; Redmond et al. 1986). Finally, CSF concentrations of HVA, 5-HIAA, and MHPG have been positively associated with ADHD symptoms (Castellanos et al. 1994).

In summary, findings suggest that CSF monoamine metabolites are state markers of elevated and depressed mood, as well as attention-deficit or hyperactivity symptoms. Much less is known about CSF monoamine metabolite status as a trait marker in clinically recovered patients. Euthymic bipolar patients have a high likelihood of relapse, even though they do not suffer from acute mood symptoms. A single study that included 25 euthymic patients with bipolar disorder and 30 healthy controls found no group differences in CSF monoamine metabolite concentrations (Berrettini et al. 1985). However, in a previous study, we found lower concentrations of HVA and 5-HIAA in bipolar disorder patients with comorbid ADHD as compared to patients with pure bipolar disorder (Ryden et al. 2009).

The aim of this study was to investigate whether CSF monoamine metabolite concentrations differ between: (a) patients with bipolar disorder and healthy controls, (b) patients with ADHD and healthy controls, and (c) patients with bipolar disorder and patients with ADHD. We thus sampled CSF from three groups: mood-stable adults with bipolar disorder, adults with ADHD, and population-based healthy controls.

## Methods

The present study is part of the St. Göran project, which provides assessment, treatment, and follow-up of patients with bipolar disorder and ADHD within the Northern Stockholm Mental Health Service. The inclusion criteria for individuals with bipolar disorder have previously been outlined in detail (Ekman et al. 2010). The key clinical assessment instrument used was the Affective Disorder Evaluation (ADE), which was developed for the Systematic Treatment Enhancement Program of Bipolar Disorder (STEP-BD) (Sachs et al. 2003). In addition to the ADE, the structured psychiatric interview, Mini International Neuropsychiatry Interview (M.I.N.I.) (Sheehan et al. 1998), was completed at baseline to screen for other psychiatric diagnoses than bipolar disorder. In addition, the Alcohol Use Disorders Identification Test (AUDIT) and Drug Use Disorders Identification Test (DUDIT) were also used to screen for substance abuse disorder. The full diagnostic assessment was based on all available sources of information, including patient interviews, case records, and, if possible, interviews with the next of kin. The diagnoses were established at diagnostic case conferences where all information available at the time of enrollment was presented. A consensus panel of experienced board certified psychiatrists specialized in bipolar disorder made a best-estimate diagnostic decision. To be included, patients were required to be 18 years or older and to meet DSM-IV criteria for bipolar spectrum disorders (i.e., type I, type II, or not otherwise specified). We collected information on age; sex; number of lifetime manic, hypomanic, depressive, and total episodes; duration of illness, defined as years since first hypomanic, or manic episode; age at onset of illness, defined as age at first hypomanic or manic episode; family history of bipolar disorder (first- or second-degree relatives with bipolar disorder); years of education; primary source of income; body mass index (BMI); and previous psychotic episodes. The severity of bipolar disorder was rated using the Clinical Global Impression (CGI) rating scales and Global Assessment of Functioning (GAF). For ethical reasons, patients continued to take their prescribed medications at the time of CSF and blood sampling.

Subjects with ADHD were enrolled from a tertiary care outpatient unit specialized in assessment and treatment of ADHD and related syndromes. One board certified psychiatrist (E.R.) interviewed all cases according to a structured intake-interview. This protocol builds on the ADE used in bipolar disorder but has been complemented with DSM-IV diagnostic criteria for ADHD. In addition to the anamnestic interview, the Wender Utah Rating Scale (WURS) (Ward et al. 1993) was used to assess childhood ADHD symptoms, and the Adult ADHD Self-Report Scale (ASRS) (Kessler et al. 2005) and Brown Attention-Deficit Disorder Scales (BADDS) (Rucklidge and Tannock 2002) were used to assess adult ADHD symptoms.

Population-based controls were randomly selected by Statistics Sweden (SCB) and contacted by mail. A research nurse contacted individuals who volunteered to participate. Eligible individuals were scheduled for a 1-day comprehensive assessment. Of the controls who received the invitation, 14% contacted the research team. This is on par with other studies of similar nature according to SCB (personal communication). Controls underwent a psychiatric interview by experienced clinicians using the M.I.N.I. to exclude psychiatric disorders. We screened for substance abuse in several ways: during the telephone interview, during the psychiatric in-person interview, using the Alcohol Use Disorders Identification Test (AUDIT) and Drug Use Disorders Identification Test (DUDIT), and by determining serum concentrations of carbohydrate-deficient transferrin (CDT). Overconsumption of alcohol, as revealed by CDT or responses indicating large consumption (>8 standard drinks per time more than twice per week) and/or amnesia and/or loss of control more than once per month, resulted in the exclusion of these individuals from the study. Other exclusion criteria were neurologic conditions other than mild migraines, untreated endocrinological disorders, pregnancy, dementia, recurrent depressive disorder and suspected severe personality disorders (based on a psychiatric interview and assessment with the Structured Clinical Interview for DSM-IV Axis II Personality Disorders), and a family history of schizophrenia or bipolar disorder in the first-degree relatives.

### CSF sampling

Bipolar disorder patients were not experiencing any acute mood episode at time of blood and CSF sampling. CSF was sampled by means of a lumbar puncture conducted between 9 and 10 am after an overnight fast. We collected a total volume of 12 mL CSF and gently inverted it to avoid gradient effects. CSF samples were divided into aliquots that were stored at −80 °C pending analysis at the Biobank at Karolinska Institutet, Stockholm, Sweden. Cases and controls underwent an identical procedure.

### Analysis of monoamines

Determination of the monoamine metabolites was performed by means of high-performance liquid chromatography with electrochemical detection, as described by Blennow et al. (1993). Intra- and inter-assay coefficients of variation were below 5% for all three analytes.

### Study population

Study data were selected from the St. Göran research database, which is an SQL-based database hosted by Gothenburg University. Data were extracted in January 2016 when the database contained information on 337 patients with bipolar disorder, 91 patients with ADHD, and 116 controls recruited in Stockholm. The inclusion criteria for this study were that each individual had to have data on monoamine metabolite concentrations and anamnestic information. For bipolar disorder, all diagnostic sub-groups with a verified bipolar disorder diagnosis were included, whereas individuals with schizoaffective disorder were excluded. In a final step, all patients with bipolar disorder with evidence of ADHD comorbidity were also excluded. Here, ADHD comorbidity was defined as a past or current M.I.N.I diagnosis, a lifetime ADHD diagnosis as defined in our previously published studies (Ryden et al. 2009), or current central stimulant treatment. This left 103 patients with bipolar disorder, 72 patients with ADHD, and 113 controls. Data on CSF monoamine concentrations for a subset of bipolar disorder patients and controls from the Stockholm center have been presented previously (Ryden et al. 2009; Sellgren et al. 2015).

### Statistics

SPSS v 20.0 was used for all statistical analysis except the power analysis (G*power v 3.1.9.2). Chi-square and ANOVA tests were used for comparisons between the three study groups, followed by Bonferroni post hoc tests. Independent sample *t* tests were used for bivariate group comparisons of continuous outcome variables. Levene’s test was used to test equality of variance across groups and non-homoscedastic variables were log^10^-transformed prior to analysis. Corrections for multiple tests were performed using the Bonferroni method. A power analysis indicated that the size of the study groups would give a power of 0.95 for detecting medium-sized effects in the between-group comparisons. For within-group comparisons, the power analyses indicated that only large effects would be reliably detectable. In all analyses, a two-sided *p* value of less than 0.05 was regarded to be statistically significant.

### Ethics

The Regional Ethics Committee in Stockholm approved our study (2005/554-31/3), which we conducted in accordance with the latest Helsinki Protocol. All patients and controls consented orally and in writing to participate in the study.

## Results

### Sample characteristics

The previous work has shown that age, sex, and body height can influence the concentrations of CSF monoamine metabolites (Blennow et al. 1993). The distributions of age, body height, and sex for the patient and control groups are shown in Table 1. One control subject had an MHPG concentration >3 standard deviations above the group mean and this observation was regarded as an outlier and excluded from all subsequent analysis. Since the three groups did not differ regarding age, sex, and body height, subsequent group comparisons were performed without correction for these covariates.

**Table 1:** Characteristics of the study population

	Control group (*N* = 113)	Bipolar disorder group (*N* = 103)	ADHD group (*N* = 72)	Statistic	*p* value
				***F***	
Age^a^	38 (13)	40 (13)	35 (10)	2.3	0.10
Height (m)^a^	1.75 (0.10)	1.73 (0.10)	1.75 (0.90)	0.8	0.46
				***χ*** ^**2**^	
Sex (female/male)	61/52	60/43	34/38	2.2	0.33

^a^Data presented as means (standard deviation)

### Monoamine metabolite concentrations and diagnosis

**Fig. 1:**
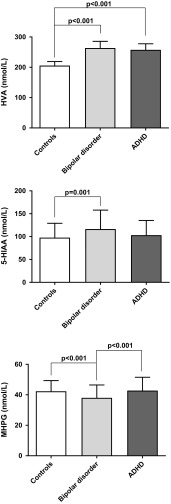
Column graph showing the CSF concentrations (nmol/L) of HVA, 5-HIAA, and MHPG in patients and controls (mean and 95% confidence interval). One-way ANOVAs were used to analyze group differences and log-transformed data were used for HVA and 5-HIAA. Bonferroni post hoc tests were conducted following significant ANOVAs to assess between-group differences (*p* values shown in graph)

Group comparisons of CSF monoamine metabolite concentrations are shown in Fig. 1. The bipolar disorder group had significantly higher HVA (264 ± 112 nmol/L, *p* < 0.001, ANOVA with Bonferroni post hoc test) and 5-HIAA (116 ± 42 nmol/L, *p* = 0.001, ANOVA with Bonferroni post hoc test) concentrations, than the control group (206 ± 70 and 98 ± 31 nmol/L, respectively). Furthermore, the ADHD group demonstrated significantly higher HVA (240 ± 94 nmol/L, *p* < 0.001, ANOVA with Bonferroni post hoc test) but not 5-HIAA (103 ± 32 nmol/L) concentrations as compared to the control group. In addition, patients with bipolar disorder had lower MHPG (38 ± 8 nmol/L, *p* < 0.001, ANOVA with Bonferroni post hoc test) concentrations than both healthy controls (42 ± 7 nmol/L) and patients with ADHD (43 ± 9 nmol/L). Finally, we recently found increased CSF concentrations of HVA in 35 euthymic bipolar patients with a history of psychosis, while the 37 bipolar patients without such a history did not differ significantly from controls (Sellgren et al. 2015). This effect was also evident here using an independent sample *t* test to compare patients bipolar disorder with and without a history of psychosis (unpaired *t* test, *t* = 3.8, *p* < 0.001). After exclusion of all patients with a history of psychosis, the difference between patients with bipolar disorder and healthy controls was no longer statistically significant (unpaired *t* test, *t* = 1.4, *p* = 0.16). Given that only one patient in the ADHD group had a history of psychotic symptoms, we did not perform the same analysis for that cohort. The clinical characteristics of the two patient groups are shown in Table 2.

**Table 2:** Clinical characteristics of the patient groups

	Bipolar disorder	ADHD
Subdiagnosis (BPI/BPII/other^a^)	55/37/11	–
Attention deficit^b^	–	62 (93)^b^
Hyperactivity^b^	–	53 (86)^c^
	***N*** **(%)**	
History of suicide attempt	36 (36)^c^	16 (28)^d^
History of psychosis	53 (52)^e^	1 (1)^f^
History of violence to others	20 (20)^e^	7 (13)^g^
History of anxiety disorder	30 (29)^e^	28 (41)^h^
History of addictive disorder	32 (32)^c^	6 (9)^h^
	**Mean (SD)**	
MADRS	6 (6)^i^	–
YMRS	1 (1)^j^	–
Lifetime number of manic episodes	2 (3)^e^	–
Lifetime number of hypomanic episodes	5 (9)^e^	–
Lifetime number of depressive episodes	11 (14)^c^	–
Lifetime number of mixed episodes	1 (4)^e^	–
GAF_function_	67 (12)^e^	65 (9)^e^
Pharmacological treatment	***N*** **(%)**	
Lithium	60 (58)	–
Lamotrigine	21 (20)	1 (1)
Valproate	14 (14)	–
SSRI	23 (22)	13 (18)
SNRI	9 (9)	11 (15)
Other antidepressant	16 (16)	2 (3)
Central stimulant	–	59 (82)
Antipsychotic	29 (28)	2 (3)

^a^A bipolar disorder diagnosis but not fulfilling criteria for type I or II. ^b^ Fulfills ASRS-based criteria for A1 and A2, respectively. Missing data for ^c^ two, ^d^ eleven, ^e^ one, ^f^ five, ^g^ eighteen, ^h^ four, ^i^ nineteen, and ^j^ twenty individuals, respectively

### Medication

To reduce the risk of false positive associations, only associations that have been previously reported were tested. For HVA, these included putative associations with lithium (Fyro et al. 1975), valproate (MacMillan et al. 1987), central stimulant (Castellanos et al. 1996), and antipsychotic (Nikisch et al. 2010; Scheepers et al. 2001) treatment. For 5-HIAA, associations with lithium (Fyro et al. 1975), valproate (MacMillan et al. 1987), and antidepressant (Backman et al. 2000) treatment were explored. Finally, for MHPG, associations with lithium (Swann et al. 1987), central stimulant (Elia et al. 1990), and antidepressant (Backman et al. 2000) treatment were tested. The effect of antidepressant treatment was tested using SSRI:s, SNRI:s, and other antidepressants as individual fixed factors in a general linear model. All other treatment effects were assessed using bivariate group comparisons using independent *t* tests. The only statistically significant associations demonstrated in the bipolar disorder group were lower 5-HIAA (general linear model, *F* = 7.3, *p* = 0.008) and MHPG (general linear model, *F* = 4.8, *p* = 0.013) concentrations in individuals treated with SSRI:s. In the ADHD group, 5-HIAA concentrations were lower in persons treated with SSRI:s (general linear model, *F* = 7.1, *p* = 0.01) or SNRI:s (general linear model, *F* = 4.4, *p* = 0.04). Finally, MHPG concentrations were also lower in individuals with ADHD treated with SSRI:s (general linear model, *F* = 4.3, *p* = 0.041) or SNRI:s (general linear model, *F* = 4.8, *p* = 0.032).

### Excluding patients with SSRIs

Given the above association between SSRIs and 5-HIAA concentrations, the effect of diagnosis on 5-HIAA and MHPG concentrations was reassessed after exclusion of all patients treated with SSRIs. This analysis showed that individuals with bipolar disorder had higher 5-HIAA (mean concentration of 124 nmol/L after exclusion), significant Bonferroni post hoc test following ANOVA (*F* = 11.2, *p* < 0.001) and lower MHPG (significant Bonferroni post hoc test following ANOVA, *F* = 6.4, *p* = 0.002) concentrations compared with both healthy controls and individuals with ADHD (mean 5-HIAA concentration of 107 nmol/L after exclusion).

## Discussion

Our first finding is that patients with mood-stable bipolar disorder had higher HVA and 5-HIAA, but lower MHPG, concentrations than healthy controls. Second, patients with ADHD had higher HVA concentrations than controls. Third, the monoamine metabolite profile in bipolar disorder differed from ADHD: Whereas both patient groups demonstrated higher HVA than controls, MHPG was significantly lower in bipolar disorder compared with ADHD.

The strengths of the present study include a relatively large sample size and well-defined and distinct patient groups. We were also able to control for a number of confounding factors that hampered the previous studies. Specifically, we addressed whether altered CSF concentrations of monoamine metabolites are trait markers of bipolar disorder or rather related to mood state or medication effects. Furthermore, we included randomly sampled population-based control groups that were collected as a part of the same study and followed the same protocol as the patient groups. However, there are also some limitations to consider. The patient cohorts were collected in a naturalistic setting where they for ethical reasons continued to take their prescribed medications. Although we accounted for medication effects in the analyses, it cannot be excluded that there are cocktail or chronic effects of psychotropic medication that we cannot dissect out. Finally, the findings of our study are limited by the fact that we have only measured metabolites and not substrates nor additional metabolites such as 3,4-dihydroxyphenylacetic acid. However, a recent study has confirmed a correlative pattern between monoamine substrates and metabolites in brain tissue (Dellu-Hagedorn et al. 2017). In the ADHD group, childhood ADHD symptoms were assessed retrospectively using the anamnestic interview and WURS scales. Thus, it is possible that this group also includes cases of adult onset ADHD. Furthermore, intelligence quotient scores were not available for the study population.

### Bipolar disorder

Patients with bipolar disorder featured higher CSF concentration of HVA and 5-HIAA, but lower concentration of MHPG. A previous study investigating CSF monoamine metabolite concentrations in mood-stable bipolar disorder patients found no difference as compared with a healthy control group (Berrettini et al. 1985). Given the relatively low number of study subjects in that study (*N* = 55) compared to ours (103 bipolar disorder subjects and 113 controls), it may, however, have been underpowered. However, the validity of our findings would be strengthened by an independent replication study.

In a previous study that included a subset of the patients in the Stockholm cohort, we found higher HVA concentrations only in mood-stable patients with a history of psychosis as compared to controls (Sellgren et al. 2015). We thus confirm this observation using a larger data set. In the study by Sellgren and co-workers, the increased HVA concentration in patients with a history of psychosis was suggested to be secondary to a kynurenic acid driven activation of the dopamine system. The association between HVA and psychotic symptoms is also supported by pharmacological evidence. Whereas dopamine agonists can trigger psychosis, a number of dopamine antagonists are effective against psychotic symptoms.

We found a low CSF concentration of MHPG in mood-stable bipolar patients, which is in line with a recent study that found low MHPG concentration in blood during remission in individuals with bipolar disorder (Kurita et al. 2014). Interestingly, other studies of acute mania (Swann et al. 1994) or atypical depression (Redmond et al. 1986) have demonstrated high MHPG concentrations in CSF in bipolar patients. Peripheral and central NE and MHPG concentrations have been found to be highly correlated (Kopin 1985), suggesting that sympathetic activity is an important determinant of CSF MHPG concentrations, which might, hence, be a state marker of mood episodes in bipolar disorder.

### ADHD

There is limited previous work on CSF monoamine metabolite concentrations in adult ADHD. One study reported no difference in CSF HVA, 5-HIAA, and MHPG between attention-impaired children and a pediatric reference group (Cohen et al. 1977). There is some evidence of a correlation between monoamine metabolite concentrations and measures of aggression and impulsivity/hyperactivity (Castellanos et al. 1994). Furthermore, the same authors have shown HVA concentration to correlate with response to stimulant treatment (Castellanos et al. 1996). Our findings suggest that remitted adult individuals with ADHD show a measurable difference in dopamine metabolism as compared to healthy controls. One previous study has found high HVA concentration in ADHD patients that were non-responders to stimulant treatment (Reimherr et al. 1984). We found no relationship between monoamine metabolite concentrations and pharmacological treatment. Our power analysis indicated that only large effects would be reliably detected in the medication sub-groups. Thus, there may be small or medium effects of medication that our study is not powered to detect. Furthermore, since only one patient in the cohort had a history of psychotic symptoms, the high HVA concentrations cannot be attributed to psychotic symptoms as suggested for the patients with bipolar disorder.

Using a sub-group of the present sample, we have previously shown an association between low HVA and 5-HIAA concentrations and childhood ADHD in bipolar I disorder (Ryden et al. 2009). In this larger sample, where individuals with comorbid ADHD were excluded, both similarities and differences were shown between bipolar disorder and ADHD. Whereas HVA concentrations were increased in both patients groups, 5-HIAA concentrations were higher only in the bipolar disorder group. The interpretation of 5-HIAA concentration might be confounded by SSRI treatment, but the exclusion of patients treated with SSRI:s confirmed the initial analysis. Furthermore, the observation of low MHPG concentration in the bipolar disorder group was not shown for ADHD. This indicates a differential involvement of monoaminergic systems in bipolar disorder and ADHD.

### Medication

SSRI treatment was linked to lower concentration of 5-HIAA in all patient groups in our study and this observation is in line with the previous clinical (Asberg and Wagner 1986; Nikisch et al. 2004; Walinder et al. 1981) and animal studies (Honig et al. 2009).

CSF monoamine metabolite concentrations have been used as indicators for monoamine turnover rates in the brain (Oreland et al. 1981). However, changes in monoamine metabolite concentrations are not readily translated into specific functional changes in monoamine signaling. Indeed, the bulk of detectable HVA and MHPG may be attributed to vesicular leakage rather than to activity dependent release (Eisenhofer et al. 2004). A previous study has also shown that cerebrospinal fluid concentrations of HVA and 5-HIAA are positively correlated to cerebral energy metabolism in patients with mood disorders (Agren and Niklasson 1988).

## Conclusion

The monoamine theory links changes in monoamine function to depressive and manic symptoms in bipolar disorder, and to attention-deficit and hyperactivity in ADHD. It has been suggested that such changes can be monitored using CSF measures of monoamine metabolites as proxy markers. In the largest study to date comparing healthy controls and individuals with bipolar disorder and ADHD, we show differences in CSF monoamine metabolites concentrations during symptom remission. Thus, altered monoamine metabolite concentrations appear to be markers of a trait dysfunction in brain monoaminergic systems.
